# An Online Platform to Support the Network of Caregivers of People with Dementia

**DOI:** 10.1155/2017/3076859

**Published:** 2017-08-15

**Authors:** April B. C. G. Boessen, Renée Verwey, Saskia Duymelinck, Erik van Rossum

**Affiliations:** Centre of Expertise for Innovative Care and Technology (EIZT), Zuyd University of Applied Sciences, Heerlen, Netherlands

## Abstract

Increasing numbers of persons with dementia (PWD) augment the pressure on dementia care, especially informal care. Care technology can support the network of PWD. We tested the usability and perceived value of an online platform that aims to support the communication and collaboration between family and professional caregivers of PWD. A mixed methods design was used for this pilot study, including semistructured interviews, a postal questionnaire, and monitoring of log data. Seven family and thirty-two professional caregivers involved with four PWD participated during a 10-week period. Overall, the results indicate that the platform is easy to use and valuable for both family and professional caregivers. They felt better informed and prepared regarding the situation of the PWD and felt supported by the more direct lines of communication within the network. Also, a broadening and deepening of the relationship between family and professional caregivers was experienced. Although connecting care organizations' record systems with the platform and an active contribution of all care professionals involved (e.g., general practitioners and those working at day care units) were suggested for optimal use of the platform, family and professional caregivers positively valued the platform for improving the efficiency and ease of communication and collaboration.

## 1. Introduction

Worldwide, the number of persons with dementia (PWD) is 47.5 million [1], of which 260,000 are living in the Netherlands [2]. Due to aging of the population, the Dutch number of PWD is expected to increase to more than half a million in 2040 [3]. This increase, as well as changes in healthcare policy towards aging in place, has a substantial impact on dementia care. For example, as elderly people, including PWD, are supposed to live longer in their home environments, psychogeriatric care in nursing homes is focused on people with advanced stages of dementia [3]. Thus, dementia care transfers from the formal (professional) to the informal (family) network of caregivers. Currently, 70% of PWD in the Netherlands live at home, of which approximately 60% reside with a partner or other family members [4]. Family caregivers residing with the PWD experience the highest burden [5]. PWD without a partner are dependent on their children, friends, and neighbours; however, not all PWD have such social networks. The Dutch Health Care Inspectorate is concerned about the neglect of PWD without a widespread social network and the overburden of the family caregivers involved [6]. Therefore, the challenge is to strengthen the network of caregivers of PWD in such a way that collaboration between formal and informal caregivers is optimally supported.

Care technology can support these networks [3]. Technologies such as sensors or GPS systems support family caregivers, increase their perceived safety, and have the potential to increase the autonomy of PWD and their ability to live independently [7–9]. Other technology-driven interventions are being developed to inform caregivers of PWD alongside behavioural change support [10]. Despite the availability of such technologies, the use of care technology is still limited in daily practice [11, 12]. Technologies are often directed at specific target groups, for example, either the formal or informal network, however, not at both [12]. In addition, different disciplines of professional caregivers use their own record systems, which fragmentizes information supply. Furthermore, professionals are often insufficiently informed about the possibilities of care technology and find it difficult to incorporate such technologies into their daily work routines [11].

Most importantly, problems are encountered in adjusting technologies to the needs and wishes of end users [13]. Verwey et al. [14] investigated the needs of family and professional caregivers in their support for PWD and the possible role of technology therein. Family caregivers specifically stated the need for good information regarding dementia and contact with fellow family caregivers [14]. Professional caregivers primarily experienced problems in sharing information regarding the situation of the PWD (e.g., changes in medication, weight, and hospitalization). They also stated that they needed clarity on the roles and responsibilities of everyone involved with the PWD. A comprehensive record system, available for all persons involved, would be helpful [14]. Based on the possible advantages of care technology in meeting these needs, an online platform was developed to support collaboration and communication between family and professional caregivers of PWD. We tested the usability and perceived value of this platform by family and professional caregivers of PWD in a pilot study.

## 2. Materials and Methods

### 2.1. Design

An iterative, user-centred design method was applied for the development of the online platform [13]. In this subsequent pilot study, a mixed methods design including semistructured interviews, a postal questionnaire, and monitoring of log data was used to evaluate the usability and perceived value of the platform [15]. The study was approved by the medical ethical committee of Zuyderland-Zuyd (15-N-207).

### 2.2. Participants

Participants in this study were family and professional caregivers of PWD. Professional caregivers of two community care organizations in Limburg, The Netherlands, recruited family caregivers of four PWD. Purposeful sampling was used in this recruitment [15], aimed at sufficient heterogeneity in the sample regarding relationship to the PWD (partner, child, etc.), residing with the PWD or not, stage of dementia, and the number of persons involved in the network of the PWD. Furthermore, family caregivers were only included when they had a sufficient level of experience with technologies such as smartphones and tablets. All professional caregivers involved with the four selected PWD were recruited, even those who were not working within the abovementioned home care organizations, such as general practitioners (GPs) and physiotherapists.

### 2.3. Materials

**Figure 1:**
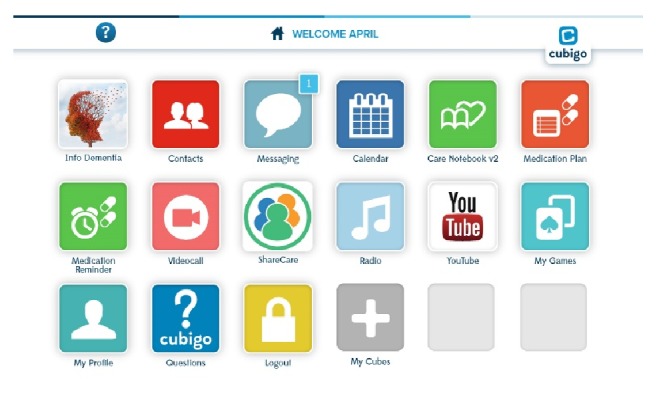
Platform Cubes for family caregivers.

Two care organizations and four ICT companies integrated their services in an online platform, based on the standard product of one of the companies. This product is an easy-to-use platform with individually tailored Cubes™ (i.e., applications) that access a range of services [16]. Services of the other companies were a video conferencing tool [17] and a tool to share care activities among (family) caregivers [18]. At the start of the study, the integrated platform consisted of standard Cubes (e.g., “Contacts/Clients,” “Messaging,” and “Calendar”), Cubes for care related information (e.g., “Info Dementia,” a care record called “Care Notebook,” “ShareCare,” and “Medication plan/reminder”), and, only for family caregivers, Cubes for entertainment (“Radio,” “YouTube,” and “My Games”). After four weeks, a “Videocall” service was added to the platform of both family and professional caregivers. An example of the platform Cubes for family caregivers is shown in Figure 1. Participants gained access to the platform via personal login codes and could individually rearrange, add, or delete Cubes according to their personal wishes.

### 2.4. Procedure

Selected family and professional caregivers received information letters on the study, including an informed consent form, which they signed before the start of the study. Prior to the start of the study, all participants received 2 h of instruction about the use of the platform. Family caregivers were instructed by the researchers and professional caregivers by an employee of the company that provided the standard platform. Furthermore, all primary family caregivers received a tablet for use during the study. Participants were also informed about the possibility of continuing to use the platform after the study.

### 2.5. Measurements

A 10-week test period took place from February to April 2016. During this period, the use of the platform was monitored by quantitative log data (i.e., clicks per Cube). Researchers did not have access to the contents of messages or the care record. The usability of the platform was measured by the Post-Study System Usability Questionnaire (PSSUQ) at the end of the test period. The PSSUQ is a validated, 19-item questionnaire that measures perceived user satisfaction with products or systems [19]. The 19 items of the questionnaire are divided over three subscales: system usefulness (8 items), information quality (7 items), and interface quality (4 items). All items were answered by using a 7-point Likert scale ranging from 1 (strongly disagree) to 7 (strongly agree). Higher scores indicate higher acceptance and usability. Furthermore, after 4 and 10 weeks, family caregivers were interviewed for approximately 1 h in their homes or another preferred location about the usability and perceived value of the platform. Professional caregivers were interviewed after 10 weeks. The development of the topic lists (Table 1) was based on the guidelines of the Dutch Institute for Healthcare Improvement [20]. During the interviews, family and professional caregivers were also asked to rate their technology experience on a 5-point Likert scale ranging from 1 (no technology experience) to 5 (excellent technology experience). Family and professional caregivers reported their experiences on feedback forms during the study as well.

### 2.6. Data Analyses

The semistructured interviews with family and professional caregivers were recorded and transcribed manually. These qualitative data, together with data from the feedback forms, were separately analysed by two researchers (AB, RV) using Nvivo (version 11) and based on the directed content analysis according to Hsieh and Shannon [21]. The initial coding scheme was determined on the basis of the Unified Theory of Acceptance and Use of Technology (UTAUT) model and was established by a consensus between the two researchers [22]. This model outlines seven main constructs that have a direct impact on behavioural intent and use of technology (performance expectancy, effort expectancy, social influence, facilitating conditions, hedonic motivation, price value, and habit). If necessary, initial codes were removed, integrated, or separated. General themes emerged, which were summarized. Data from the PSSUQ questionnaire were analysed using descriptive statistics and *t*-tests in SPSS (version 23), and log data were analysed using Excel 2016.

## 3. Results

In total, 7 family caregivers and 32 professional caregivers participated in this study. These participants were involved in the care of four PWD. Participant characteristics are shown in Table 2.

### 3.1. Use of the Platform Based on Log Data

**Figure 2:**
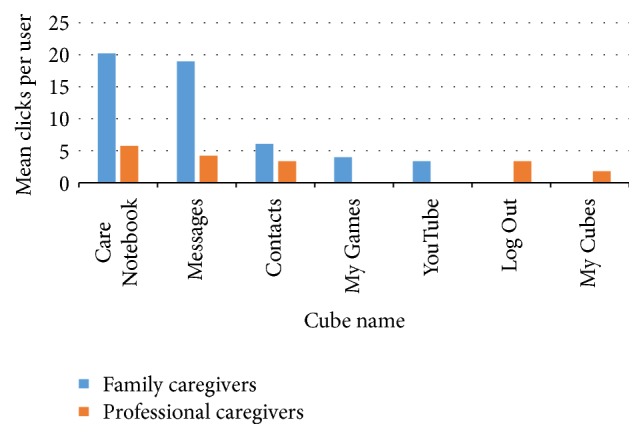
Mean clicks per user of the five most frequently used Cubes.

Only a few Cubes were frequently used by both family and professional caregivers. Mean clicks per user of the five most frequently used Cubes are shown in Figure 2. The care record and message Cubes were most frequently used by both groups of caregivers. Use of these Cubes was three to four times higher for family caregivers compared with professionals. Clicks per user per week (not shown) indicated that use of these Cubes by family caregivers was the highest in the second half of the study, whereas the use of these Cubes was the highest in the first weeks and stabilized afterwards for professionals. In contrast to these Cubes, use of “Contacts” was much lower among family caregivers, but comparable to its use among professionals. Both family and professional caregivers used “Contacts” mainly at the start of the study. Family caregivers also used the Cubes “YouTube” and “My Games” (only with a peak in one of the weeks). Among professional caregivers, “Log Out” and “My Cubes” were in the top five of most frequently used Cubes. Follow-up log data showed that 71% of family caregivers and 31% of professional caregivers continued using the platform for up to 10 weeks after the test period and a similar proportion of family caregivers and 15% of professional caregivers continued using it for up to 20 weeks after the test period.

### 3.2. Use of the Platform Based on Interviews

Most family caregivers reported daily use of the platform, usually on a laptop or tablet. Professionals reported using the platform on a weekly basis or more frequently if needed in view of the complexity of the client's situation. They preferred using a tablet or smartphone. With “use of the platform,” both groups mainly referred to its care record and message functions. These were perceived as the most important Cubes to communicate with others:* “Via the care record and message functions, I share information about the situation of my partner and reach every person involved” (network 2, son, 63 years)*. Some family caregivers mentioned using the Cubes “Calendar” and “Info Dementia” in the beginning, but this was not continued in most cases. Most of the other Cubes were perceived as unnecessary or their functions were not understood by both family and professional caregivers:* “I did not use the medication reminder. I did not understand what to do with it. Is it a kind of alarm? Or is it only useful if the client has access to the platform as well?” (network 4, daughter, 56 years).*

### 3.3. Usability Measured by the PSSUQ

Usability of the platform was rated with an average score of approximately 6 per item (data not shown) as well as per subscale (Table 3). Only a mean score of 3.3 for the item “The platform gave error messages that clearly told me how to fix problems” (system usability subscale) was relatively low. On average, family caregivers scored this item with a 2.0 and professionals caregivers with a 3.4. Scores between family and professional caregivers significantly differed for only one item on the system usability subscale: “It was simple to use this platform” (*χ*^2^ (2, *n* = 26) = 5.085, *p* = 0.034), which professionals rated more positively (mean of 6,4) than family caregivers (mean of 5,7).

### 3.4. Perceived Value Based on Interviews

Overall, both family and professional caregivers were optimistic about the use of the platform. Soon after the start, participants indicated that they would like to continue using the platform after the study. Family caregivers felt that they were more easily and quickly informed about the situation of the PWD:* “On the days that I am not able to visit my father, I now know someone else did and the platform makes it able to read what this person discussed or undertook with my father or noticed about him. In the current situation, this person would never call to inform me” (network 3, daughter, 38 years)*. Professionals perceived shorter communication lines with other professionals as well as family caregivers and therefore could take action more quickly. This was mainly seen as an advantage for more complex cases, where communication was essential. Due to this improved information provision, family caregivers felt calmer and more prepared before or without visiting the PWD. Professionals added that they had an overview of the network involved with a particular client:* “The advantage of the platform is that we as professionals are more aware of situations that occur regarding the client and of the persons involved with this client” (network 4, community nurse, 65 years)*. Furthermore, family caregivers felt supported by the direct communications lines with professionals and their increased involvement. Professionals also perceived a broadening and deepening of the relationship with family caregivers:* “I think the relationships with families of clients have broadened in a certain way. There is more involvement and understanding of the situation on both sides” (network 1, nurse, 54 years)*. This increased involvement was recognized by the fact that professionals reported using the platform outside working hours or at home. Family caregivers reported that it was sometimes difficult to discuss problems with others in the presence of the PWD. Therefore, they were optimistic about the possibility of using the platform to communicate about the PWD without causing commotion or confusion for the PWD.

### 3.5. Suggestions for Improvement Based on Interviews

Both family and professional caregivers advised limiting the number of Cubes, so that only Cubes with added value for family and professional caregivers would be available:* “The platform is a system with lots of different services of which I think not all are necessary. Keep it simple!” (network 4, daughter, 59 years)*. A challenge for professionals was that they had to continue using their current record systems, while reporting similar information in the care record of the platform. For them, the ideal situation would be a connection between the care organizations system and the platform. Family caregivers thought that professionals could have used the platform more frequently, although they mentioned that the dual recording was a possible reason for their disappointing frequency of use:* “The double records that professionals had to write might have discouraged them from using the platform more often” (network 2, son, 63 years)*. Along these lines, some family caregivers mentioned that they had to take the first step in using the platform, although they thought that professionals could have taken this initiative as well. However, when the lines of communication were established, communication was initiated both ways. For optimal information provision and thus use of the platform, both family and professional caregivers thought it was essential to include every person involved in the care of the PWD:* “Every person who visits the client could provide important information to this online network, even an alert domestic worker who might notice the client's falls” (network 4, physiotherapist, 56 years)*. Furthermore, some technical issues of the platform arose, such as starting problems (e.g., white screen) and a high battery consumption of the application on smartphones.

## 4. Discussion

The results of this pilot study show that family and professional caregivers of PWD find the online platform easy to use and valuable. They indicated that the platform supported better information provision regarding the situation of the PWD, shortened communication lines within a caregiver network, and improved the involvement and commitment of caregivers towards each other and the PWD. Actual use of the platform appeared to be mainly restricted to the communication and care registration functionalities. Important suggestions for optimal use were connecting care organizations' record systems with those of the platform (to prevent dual recording) and active contributions of all caregivers involved (e.g., GPs and those working at day care units).

A previous needs assessment study among family and professional caregivers of PWD identified needs regarding care of PWD, which primarily concerned needs for better information provision, contact with fellow family caregivers, and a comprehensive record systems for all persons involved [14]. Participating family and professional caregivers welcomed the deployment of an online platform in meeting these needs. In this study, the technological feasibility of such an online platform is shown, as well as its perceived value among family and professionals caregivers. Other studies aimed at technological support of caregivers of PWD mainly focus on caregiver burden. Technology platforms and telecommunication or Internet-based platforms indicated a reduction of this burden [23–25]. In line with these results, we found that caregivers valued the platform for shortening communication lines between caregivers and improving caregiver involvement, which both may contribute to reducing perceived caregiver burden.

Today, many technological (care) platforms with diverse contents are being developed, which results in fragmentation as well as confusion for caregivers [26]. Different care organizations and companies develop their own platforms separately, whereas the functionalities of these platforms have many similarities. Users must log on to too many different systems. The platform in this study was developed as a response to this fragmentation with the aim of integrating various services and products so that end users would have access to various functionalities via one platform. Family and professional caregivers in this study only seem to feel a need for those functionalities supporting communication and joint care registration (and only if an active contribution of all caregivers involved would be guaranteed). This is in line with previous studies reporting that the value of (assistive) technologies mainly lies in functionalities that facilitate care coordination or communication between family and/or professional caregivers [25, 27, 28]. Although today's technological possibilities in care settings are far reaching (e.g., remote care and video calls), networks of caregivers do not really seem to miss such possibilities. Possible reasons for the limited use of some functionalities of the platform in this study might be a mismatch between family and professional caregivers' needs and technology requirements of the platform or insufficient instructions to participants regarding the use of the different functionalities of the platform. Another important factor that hampered the integration of services is that different care professionals (e.g., nurses, GPs, and physiotherapists) use different client registration systems. GPs experience the lack of possibilities to connect or integrate different applications as one of the most important constraints to the exchange of patient-related information [29]. This inability to integrate applications entails double recording of information, which seems to have influenced the frequency or content of use by the professionals in this study. Professional caregivers involved therefore advised making connections with the different record systems they use within the care registration functionality of the platform.

A strength of the study was the user-centred approach. This approach supports a feel of ownership of the final product among participants. The collaboration with the networks of the PWD resulted in a higher consumer satisfaction and could lead to a smoother implementation of the product [30]. With regard to the methodology, data triangulation increased the credibility and confirmability of this study [31]. As professional caregivers recruited family caregivers, this may have introduced some selection bias. They may have selected mainly family caregivers who were enthusiastic and willing to use the platform (e.g., early adopters or early majority) [32]. Furthermore, they only selected PWD who received care within the care organizations, although the platform might also be useful for networks of PWD with little or no professional care involved. Since this was a pilot study, we believe that the impact of these impediments upon the results was limited. The experiences of participants in this study, regardless of their early adopter status or the care situation of the PWD, show the potential of the platform.

Before technologies such as the platform in this study can be implemented on a large scale, further robust demonstration of its application should be investigated. Testing the usefulness and perceived value of the platform with a small group of caregivers, as we did in this study, is one of several steps in the iterative user-centred design process [13]. Although the results are promising and the number of participating caregivers per network supporting the PWD was high, the number of networks studied should be increased to obtain a better view on the platform's feasibility and generalizability of the results. To realize the potential of this online platform for family and professional caregivers, a number of issues should be addressed in a feasibility study. For example, how do family and professional caregivers experience an online instead of face-to-face introduction to the platform (due to time and resource restraints) and how do professional caregivers use and value the platform in case of their involvement with more than one PWD and his or her family caregivers? Therefore, we initiated a feasibility study in which the impact and potential limiting and facilitating factors for using the platform are investigated in a larger group of family and professional caregivers.

## 5. Conclusion

We showed the technological feasibility of an online platform that integrates various functionalities or applications. The results of this pilot study are promising for future use of the online platform. Use of the online platform was accepted and valued by family and professional caregivers as a result of perceived improvements in communication and collaboration regarding the care of the PWD. However, the results should still be tested on a larger scale.

## Figures and Tables

**Table 1:** Topic lists of interviews with caregivers.

Family caregivers	Professional caregivers
Actual use: frequency, duration, Cubes	Actual use: frequency, duration, Cubes
Usability: efficiency, information, interface, possibilities	Usability: efficiency, information, interface, possibilities
Key experiences: positive/negative	Key experiences: positive/negative
Support by professional caregivers	Collaboration with family caregivers (frequency and content of communication, sharing care)
Communication with family or professional caregivers	Collaboration with other professional caregivers (frequency and content of communication, sharing care)
Collaboration/sharing care	Care registration
Future use	Use at organizational levels
Finances/costs	Finances/costs

**Table 2:** Participant characteristics.

	Family caregivers (*n* = 7)	Professional caregivers (*n* = 32)
Mean age (SD)	59.2 (17.6)	44.6 (16.5)
Gender: female	5	29
Mean number of persons involved per client (SD)	1.75 (0.5)	9 (3.7)
Position in network	Daughter (*n* = 5) Son (*n* = 1) Partner (*n* = 1)	Nurse (*n* = 23) Case manager (*n* = 4) General practitioner (*n* = 1) Physiotherapist (*n* = 1) Day care professional (*n* = 1) Domestic worker (*n* = 1) Care farm worker (*n* = 1)
Technology experience^*∗*^: mean (SD)	3.7 (0.5)	3.1 (1.1)

^*∗*^Scores varied from 1 (no technology experience) to 5 (excellent technology experience).

**Table 3:** Mean scores^*∗*^ of family and professional caregivers per subscale of the PSSUQ.

	Family caregivers	Professional caregivers
System usefulness		
*N*	7	19
Mean (SD)	6,07	6,15
Information quality		
*N*	6	19
Mean (SD)	5,55	5,87
Interface quality		
*N*	5	18
Mean (SD)	6,31	6,05

Scores varied from 1 (strongly disagree) to 7 (strongly agree).
